# Ultra-High Dose Rate FLASH Irradiation Induced Radio-Resistance of Normal Fibroblast Cells Can Be Enhanced by Hypoxia and Mitochondrial Dysfunction Resulting From Loss of Cytochrome C

**DOI:** 10.3389/fcell.2021.672929

**Published:** 2021-04-30

**Authors:** Jintao Han, Zhusong Mei, Chunyang Lu, Jing Qian, Yulan Liang, Xiaoyi Sun, Zhuo Pan, Defeng Kong, Shirui Xu, Zhipeng Liu, Ying Gao, Guijun Qi, Yinren Shou, Shiyou Chen, Zhengxuan Cao, Ye Zhao, Chen Lin, Yanying Zhao, Yixing Geng, Jiaer Chen, Xueqing Yan, Wenjun Ma, Gen Yang

**Affiliations:** ^1^State Key Laboratory of Nuclear Physics and Technology, School of Physics and CAPT, Peking University, Beijing, China; ^2^Teaching and Research Section of Nuclear Medicine, School of Basic Medical Sciences, Anhui Medical University, Hefei, China; ^3^Collaborative Innovation Center of Extreme Optics, Shanxi University, Taiyuan, China

**Keywords:** ultra-high dose rate irradiation, FLASH, radio-resistance, cell death, hypoxia, normoxia, mitochondria, cytochrome c

## Abstract

Ultra-high dose rate FLASH irradiation (FLASH-IR) has got extensive attention since it may provide better protection on normal tissues while maintain tumor killing effect compared with conventional dose rate irradiation. The FLASH-IR induced protection effect on normal tissues is exhibited as radio-resistance of the irradiated normal cells, and is suggested to be related to oxygen depletion. However, the detailed cell death profile and pathways are still unclear. Presently normal mouse embryonic fibroblast cells were FLASH irradiated (∼10^9^ Gy/s) at the dose of ∼10–40 Gy in hypoxic and normoxic condition, with ultra-fast laser-generated particles. The early apoptosis, late apoptosis and necrosis of cells were detected and analyzed at 6, 12, and 24 h post FLASH-IR. The results showed that FLASH-IR induced significant early apoptosis, late apoptosis and necrosis in normal fibroblast cells, and the apoptosis level increased with time, in either hypoxic or normoxic conditions. In addition, the proportion of early apoptosis, late apoptosis and necrosis were significantly lower in hypoxia than that of normoxia, indicating that radio-resistance of normal fibroblast cells under FLASH-IR can be enhanced by hypoxia. To further investigate the apoptosis related profile and potential pathways, mitochondria dysfunction cells resulting from loss of cytochrome c (cyt c^–/–^) were also irradiated. The results showed that compared with irradiated normal cells (cyt c^+/+^), the late apoptosis and necrosis but not early apoptosis proportions of irradiated cyt c^–/–^ cells were significant decreased in both hypoxia and normoxia, indicating mitochondrial dysfunction increased radio-resistance of FLASH irradiated cells. Taken together, to our limited knowledge, this is the first report shedding light on the death profile and pathway of normal and cyt c^–/–^ cells under FLASH-IR in hypoxic and normoxic circumstances, which might help us improve the understanding of the FLASH-IR induced protection effect in normal cells, and thus might potentially help to optimize the future clinical FLASH treatment.

## Introduction

Radiotherapy (RT) makes up an essential part of cancer treatment and more than half of cancer patients receive radiotherapy during treatment (Vozenin et al., 2019a). In conventional radiotherapy, the radiation includes photons, electrons, protons and heavy ions (Valentini et al., 2020). Although major advances in precise treatment delivery and multimodal imaging allowed radiotherapy to apply to more patients, radiation resistance still remains an unsolved clinical problem, thus more powerful and better tolerated radiotherapy was needed (Vozenin et al., 2019a). A novel modality of irradiation, named FLASH radiotherapy (FLASH-RT), has got extensive attention in recent years, since it provides better protection on normal tissues while maintains tumor killing effects (Durante et al., 2018). At the biological level, the increased radio-resistance and reduced normal tissue toxicity has been named as the FLASH protection effect (Montay-Gruel et al., 2018). Compared with conventional irradiation (∼1 Gy/min), FLASH-RT provides ultra-high dose rate (>40 Gy/s) and is always delivered in an ultra-short time (<0.1 s) (Durante et al., 2018). Normal tissue sparing by FLASH of many organs has been demonstrated in previous experiments (Bourhis et al., 2019; Griffin et al., 2020). Favaudon et al. (2014) found that the pulmonary fibrosis of mouse didn’t appear at 36 weeks after FLASH irradiation while it appeared at 8 weeks after the same dose with conventional dose rate. Montay-Gruel et al. found that spatial memory of mice was preserved after whole brain irradiation with >100 Gy/s dose rate while it was totally impaired with 0.1 Gy/s dose rate at same dose (Petersson et al., 2017). Loo et al. (2017) found that when receiving abdomen irradiation between 10 and 22 Gy, the median lethal dose (LD50) of mouse is 14.7 Gy for 0.05 Gy/s dose rate and 18.3 Gy for 210 Gy/s dose rate. The FLASH IR experiments could be performed on conventional accelerators and on compact laser-driven accelerators as well (Ledingham et al., 2014). The laser-driven proton beams with duration scale of nanosecond at the irradiation site can improve dose rates to about 10^9^ Gy/s (Bin et al., 2012; Doria et al., 2012; Hanton et al., 2019). Several FLASH IR studies have been reported *in vitro* (Zeil et al., 2013; Raschke et al., 2016; Bayart et al., 2019) and *in vivo* (Rosch et al., 2020) with laser-driven protons, aiming to clarify the radiobiological effects of the high dose rate proton bunches.

Currently the exact mechanism of FLASH effect on normal tissues is not clear, and is suggested to be closely related to the oxygen consumption in tissues (Wilson et al., 2012). The hypothesis is as follows, FLASH with high total dose depletes oxygen within ultra-short time, and it is too quickly for diffusion and reoxygenation, so the normal tissue responds as hypoxic tissue (Durante et al., 2018). Therefore, ultra-high dose rate will increase the radio-resistance of the normal tissue while have small impact on the already hypoxic tumor tissue (Durante et al., 2018).

Irradiation induces cell death mainly by damaging DNA either directly or indirectly (Korystov, 1992). Among the cell death after irradiation, apoptosis is a highly regulated form with characteristic morphological and molecular features (Eriksson and Stigbrand, 2010; Sia et al., 2020). The mitochondrial apoptotic pathway is activated when the DNA damage repair machinery disrupts the balance between pro- and anti-apoptotic factors, resulting in the release of cytochrome c (cyt c) from mitochondria (Sia et al., 2020). The externalization of phosphatidylserine on the plasma membrane is a marker of early stage apoptosis (Yun and Lee, 2016) and can be detected by annexin-V (Xu and Leung, 2010; Liu et al., 2013), while late apoptosis and secondary necrosis have the feature of DNA and nuclear fragmentation and permeability of the plasma membrane to propidium iodide (Bacso et al., 2000; Troiano et al., 2007; Foldbjerg et al., 2011).

Cyt c is a pivotal protein residing in mitochondria, and acts as a component of mitochondria respiration and apoptosis initiator (Yang et al., 2009). It transports electrons from Complex III to Complex IV in respiratory chain and is crucial for ATP synthesis through oxidative phosphorylation (Yang et al., 2009; Wilson and Vinogradov, 2014). In response to stimuli such as radiation induced DNA damages, cytochrome c is released from intermembrane space of mitochondria, binds Apaf-1 with high affinity in cytosol and triggers oligomerization of Apaf-1/cyt c complexes that activate procaspase-9. And then activated caspase-9 cleaves and activates caspase-3, caspase-6 and caspase-7, which function as downstream effectors of the cell death (Li et al., 2000; Wang and Youle, 2009).

Due to its significant clinic potential of the fledging ultra-high dose rate FLASH-IR area, there are many fundamental questions need to be carefully re-visited as conventional low dose rate irradiation, especially the basic killing effect of FLASH on normal cells in hypoxic and normoxic condition. In this article, cyt c-normal (cyt c^+/+^) and -null (cyt c^–/–^) mouse embryonic fibroblast cells were irradiated and traced after FLASH-IR (>10^9^ Gy/s) at a dose of ∼10 to 40 Gy in hypoxic and normoxic condition, using CLAPA ultra-fast laser-generated particles, and the dose was determined by the experimental ion spectra and dose spatial distribution combined with Monte Carlo simulations.

## Materials and Methods

### Cell Culture

Cyt c-normal and -null mouse embryonic fibroblast cells are kind gifts from Dr. Xiaodong Wang (Li et al., 2000; Yang et al., 2009). The cells were cultured in Dulbecco’s modified Eagle’s medium (DMEM, HyClone) supplemented with 20% fetal bovine serum (FBS, Bai Ling Biotechnology), 100 U/ml penicillin and streptomycin (P/S) (HyClone), 50 mg/L uridine (Macklin), 2 mM glutamine (Boyobio), 1 × non-essential amino acids (Coolaber), 55 mM 2′-mercaptoethanol (Macklin), 2.5 M HEPES (Boyobio) and 5 × 10^5^ unit/L mouse leukemia inhibitory factor (Yize). The cyt c-null cell culture medium was added with 110 mg/L pyruvate (Psaitong) for the compensation of the defect in mitochondrial respiration. And the petri dishes for both cell lines were pre-coated with 1% geltrex (Gibco) as described previously (Fu et al., 2017) and cells were cultured at 37°C in a humidified incubator (5% CO_2_).

### Detection of Viability

The Calcein-AM/PI staining kit (BestBio) was used to assay the viability of cyt c^+/+^ and cyt c^–/–^ cells. Cells were harvested at the confluence of 70–80%, transfered to 96-well plate and stained with Calcin-AM and PI. Live cells and dead cells showed green fluorescence and red fluorescence respectively.

### Experimental Setup

**FIGURE 1:**
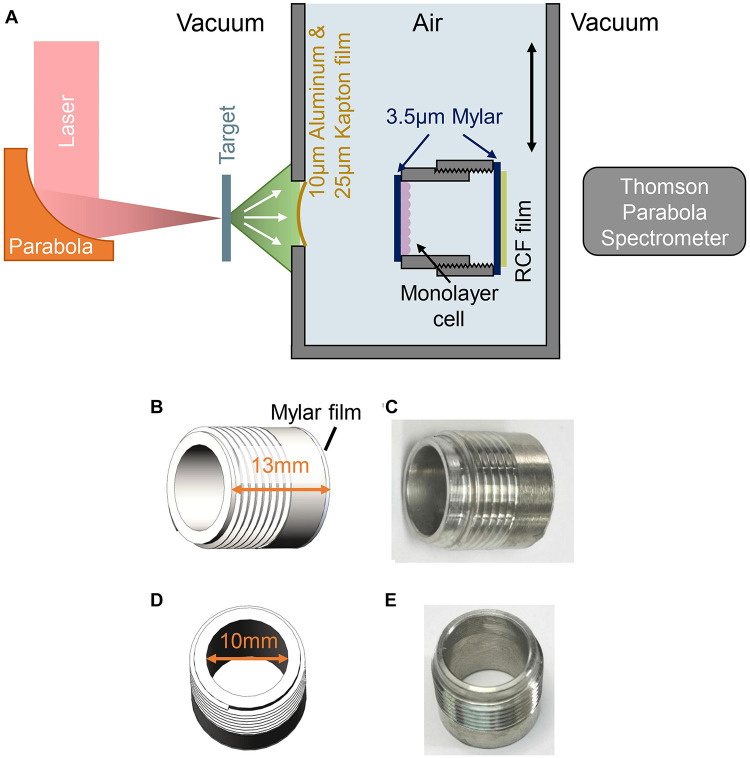
Experimental setup for FLASH irradiation. **(A)** Schematic drawing of experimental setup. **(B,C)** Side view of the cell-culture cylinder, cells were cultured on the 3.5 μm Mylar film adhered to inner side of the cylinder. **(D,E)** Top view of the cell-culture cylinder.

The FLASH radiation experiment was performed using Compact Laser Plasma Accelerator (CLAPA) system at Peking University. The CLAPA laser is a Ti: sapphire laser with central wavelength of 800 nm and full-width-at-half-maximum duration of 30 fs. A single plasma mirror system was used to increase the temporal contrast to 10^8^@5 ps. S-polarized laser was focused onto the 100 nm plastic target using an f/2.5 off-axis parabolic (OAP) with a focal length of 200 mm. The laser energy on target was ∼1 J and the diameter (FWHM) of focal spot was 4.2 μm, containing 36% of the total energy, resulting in the peak intensity of 5.6 × 10^19^ W/cm^2^. The target got broken by the laser at each shot, generating ultra-short ion bunch. The ion energy spectra were measured by a Thomson parabola spectrometer (TPS) positioned at 780 mm along the direction normal to the back surface of the target. The cells irradiation system was plugged in after the ion energy spectra were measured (Figure 1A). The ion beams entered cells radiation system through a vacuum window and irradiated the cells.

### Dose Monitoring

The ions were accelerated in well-known Target Normal Sheath Acceleration (TNSA) regime (Passoni et al., 2010), which leads to exponentially decreasing energy spectra up to a cut-off energy around 5–6 MeV depending on the on-target laser intensity. The parabolic traces of ions were obtained by the Thomson Parabola Spectrometer, recorded by a micro-channel plate with a phosphor screen and imaged onto a 16-bit EMCCD camera. After the proton energy was known and the cells irradiation system moved in, customized radiochromic film (RCF) EBT3 (the clear polyester layer facing the target was removed) was placed behind the cell sample for dose measured. To obtain the dose deposited in the cell from the measured dose in EBT3 films, the Monte-Carlo code FLUKA (Bohlen et al., 2014) was put into use.

### Irradiation Protocol

Cells were cultured on the 3.5 μm Mylar film pasted by glue at the bottom of cell cylinder for irradiation. The cell cylinder was pre-treated with 1% geltrex for 24 h and seeded cells 20 h before irradiation. Cyt c-normal and -null mouse embryonic fibroblast cells were digested and adjusted to appropriate density, and ∼120,000 cells were added to each cell cylinder. The hypoxic condition was mimicked by adding CoCl_2_ (Sigma) at the final concentration of 100 μM to the medium for 6–12 h as reported previously (Wu and Yotnda, 2011).

The medium in the cell cylinder was aspirated right before irradiation, and after irradiation the cell cylinder was supplemented with medium immediately and put into the incubator. At 6, 12, and 24 h after the irradiation, the cells on the mylar film were digested, mixed and transferred to 96-well plate for detection of apoptosis.

### Conventional Irradiation

Cyt c^+/+^ and cyt c^–/–^ cells were seeded on 24-well plate for adherence before irradiation, with ∼120,000 cells each well. Then cells were irradiated by Co-60 γ radation at 3 Gy/min for 10 min (30 Gy total doses). At 6, 12, and 24 h after the irradiation, cells were transferred to 96-well plate for detection of apoptosis by Dead Cell Apoptosis Kit with Alexa Fluor 488 annexin V and PI (Invitrogen).

### Detection of Apoptosis

To detect apoptosis of cells after FLASH radiation, we used Dead Cell Apoptosis Kit with Alexa Fluor 488 annexin V and PI (Invitrogen). Cells digested from the mylar film were added to 96-well plate with 100 μL for each well. Then 1 μL 100 μg/mL PI, 5 μL Alexa Fluor 488 annexin V and 0.2 μL Hoechst 33342 (Thermo Fisher) working solution were added to the well. Cells were stained 15 min in the incubator and then imaged by Spark^®^ Cyto automatic real-time live cell imaging detection system (TECAN). The cell nuclei are stained by Hoechst, cells with Hoechst+, annexin V+ and PI− were recognized as early apoptotic cells, while Hoechst+/annexin V+/PI+ represent late apoptotic and necrotic cells.

### Detection of Caspase-3/7

Caspase-3/7 were detected using Caspase-Glo^®^ 3/7 Assay (Promega) at 24 h after conventional irradiation. In a 96-well plate, 100 μL of cell suspension and 100 μL Caspase-Glo^®^ 3/7 Reagent were added, and the chemiluminescence was detected after 1 h. The detection was performed by Spark^®^ Cyto automatic real-time live cell imaging detection system (TECAN).

### Data Analysis

Results were expressed as means ± SD, and one-way ANOVA was used to analyze the significant differences between control and experimental group. *P* < 0.05 was marked as ^∗^, *P* < 0.01 was marked as ^∗∗^, *P* < 0.001 was marked as ^∗∗∗^. At least 3,000 cells were counted in each group.

## Results

### FLASH Irradiation Setup

The schematic of the experimental setup was shown in Figure 1A, the ion beams entered cells irradiation system through a vacuum window located 63 mm behind the target and irradiated the cells. The vacuum window is composed of a 10 μm Aluminum film for light-shielding, and a 25 μm Kapton film for vacuum sealing. Monolayer cells cultured on a 3.5 μm Mylar film were mounted on a stainless-steel cell-culture cylinder, facing to the inner space of the cylinder as shown in Figures 1C,E. The inner diameter and the height of the cylinder were 10 and 13 mm, respectively (Figures 1B,D), and the cylinder was mounted on a holder, locating the cell-cultured Mylar film vertically to receive the irradiation. The distance from the vacuum window to the cell plane is 7 mm. Another 3.5 μm Mylar film was taped on the other side of the holder to seal the cylinder, avoiding the contamination and dryout of the cell during irradiation. Each cell sample was irradiated by a single shot.

### Dose Calculation

**FIGURE 2:**
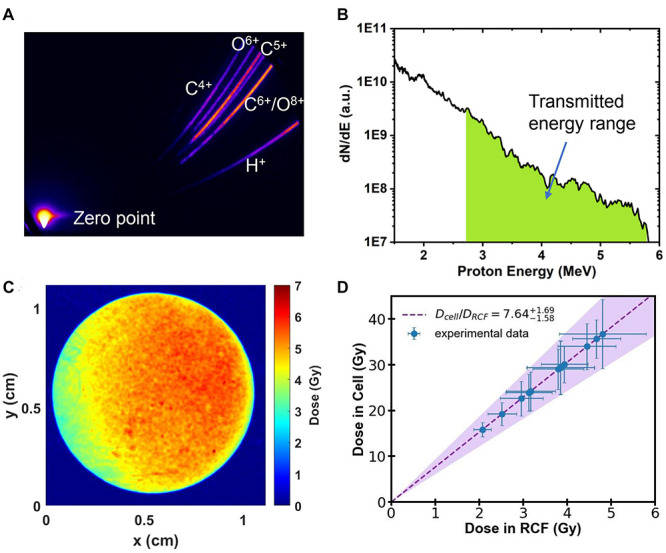
**(A)** Raw image obtained by the Thomson Parabola Spectrometer. **(B)** Typical energy spectra of laser-accelerated protons. Protons with energy in the green area can pass through the monolayer cell. **(C)** Spatial distribution of dose detected by RCF film from a single shot. **(D)** The dose deposited in the monolayer cell as a function of the measured dose with RCF film.

The parabolic traces of ions were obtained by the Thomson Parabola Spectrometer, recorded by a micro-channel plate with a phosphor screen and imaged onto a 16-bit EMCCD camera. A typical raw image of the parabolic traces measured by the Thomson Parabola Spectrometer is shown in Figure 2A. The corresponding energy spectra of protons with cut-off energy of 5.8 MeV are shown in Figure 2B. Protons in the energy range marked in green can pass through the monolayer cell and detected by the EBT3 film. Generated oxygen/carbon ions and protons lower than 2.7 MeV were completely stopped by the vacuum window and the air.

Customized EBT3 film for dose verification was placed 25 mm away from the cells, directly behind the sealing Mylar film. The irradiated EBT3 films were scanned with an Epson Perfection V700 scanner in transmission mode. The dose response of EBT3 was calibrated following the literature reported previously (Reinhardt et al., 2012). The spatial distribution of dose detected by EBT3 film in a single laser shot is shown in Figure 2C, which has RMS (root mean square) variation of 16%.

We employed the Monte-Carlo code FLUKA (Bohlen et al., 2014), input all the setup parameters and the measured energy spectra of protons, to determine the dose deposited in the cell from the measured dose in EBT3 films. The calculated dose delivered to the cell is shown in Figure 2D. The filled area shows the calculation result considering the shot-to-shot fluctuations and the error bar of the data represents the RMS variation of the dose distributions. The summary of the dose in both layers for each shot is shown in Table 1.

**TABLE 1:** Summary of laser accelerator dose for each FLASH experiment.

	**Dose in RCF film**	**SD. Of Dose in RCF film**	**Calculated average dose in cells**	**SD of dose in cells**	**Shot used**
1	3.9	0.5	30.1	4.1	cyt c^+/+^ hypoxia 6 h
2	4.8	1.0	36.8	7.6	cyt c^+/+^ hypoxia 12 h
3	4.7	0.6	35.7	4.2	cyt c^+/+^ hypoxia 24 h
4	3.2	0.6	24.2	4.3	cyt c^+/+^ normoxia 6 h
5	3.0	0.5	22.6	3.7	cyt c^+/+^ normoxia 12 h
6	3.9	0.8	29.4	5.9	cyt c^+/+^ normoxia 24 h
7	3.1	0.5	23.9	4.1	cyt c^–/–^ hypoxia 6 h
8	2.1	0.2	15.8	1.5	cyt c^–/–^ hypoxia 12 h
9	2.5	0.3	19.3	2.5	cyt c^–/–^ hypoxia 24 h
10	3.8	0.6	29.0	4.4	cyt c^–/–^ normoxia 6 h
11	3.9	0.8	29.4	5.8	cyt c^–/–^ normoxia 12 h
12	4.5	0.7	34.0	5.0	cyt c^–/–^ normoxia 24 h
13	3.5	0.5	32.1	4.7	cyt c^+/+^ hypoxia 6 h
14	4.5	0.8	41.1	7.0	cyt c^+/+^ hypoxia 12 h
15	4.4	0.6	40.9	5.9	cyt c^+/+^ hypoxia 24 h
16	3.6	0.4	33.5	4.1	cyt c^+/+^ normoxia 6 h
17	5.2	1.0	47.5	9.2	cyt c^+/+^ normoxia 12 h
18	6.1	1.6	56.0	14.4	cyt c^+/+^ normoxia 24 h
19	3.1	0.5	28.1	4.2	cyt c^–/–^ hypoxia 6 h
20	4.7	0.7	42.8	6.4	cyt c^–/–^ hypoxia 12 h
21	4.8	0.8	43.8	6.9	cyt c^–/–^ hypoxia 24 h
22	2.4	0.5	5.2	1.2	cyt c^+/+^ hypoxia 12 h
23	1.3	0.2	2.8	0.4	cyt c^+/+^ hypoxia 24 h
24	0.9	0.1	1.8	0.2	cyt c^+/+^ normoxia 12 h
25	1.0	0.1	2.0	0.2	cyc c^+/+^ normoxia 24 h
26	2.2	0.3	4.9	0.4	cyc c^+/+^ hypoxia 12 h
27	3.9	0.8	8.6	1.7	cyc c^+/+^ hypoxia 24 h
28	2.7	0.6	5.9	1.2	cyc c^–/–^ hypoxia 12 h
29	4.2	1.1	9.2	2.5	cyc c^–/–^ hypoxia 24 h

In TNSA regime, the laser acceleration is completed within 1 ps (Bayart et al., 2019). Considering the broadening of the beam duration due to the difference of the flying speed of protons, the duration of the proton pulse delivered to the cell plane was ∼10 ns, corresponding to the dose rate in the order of 10^9^ Gy/s.

### Detection of Cell Viability

As shown in Supplementary Figure 5, the survival proportion of cyt c^+/+^ and cyt c^–/–^ cells were 93.4 and 92.5%, respectively. Cyt c^+/+^ cells showed a little bit higher viability, but the difference between the two type of cells was not significant.

### Mouse Embryonic Fibroblast Cells Are More Resistant to FLASH Irradiation in Hypoxic Condition

**FIGURE 3:**
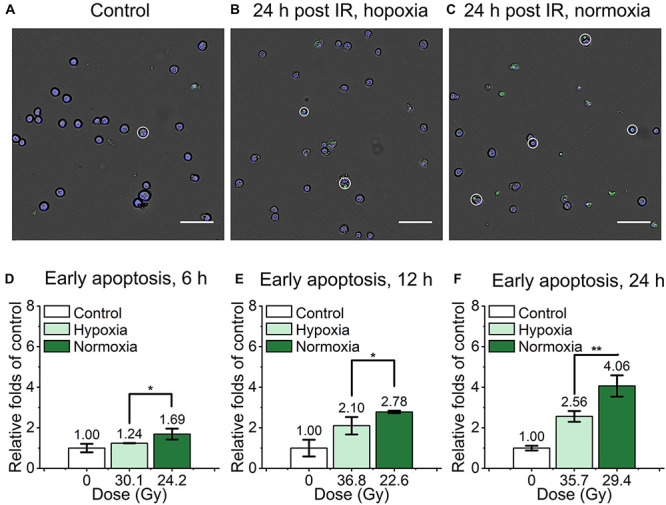
Early apoptosis of irradiated normal fibroblast cells after FLASH irradiation. **(A–C)** Representative images of control **(A)**, irradiated cells in hypoxic **(B)** and normoxic condition **(C)** at 24 h after radiation. Early apoptotic cells were marked with white circles. Scale bars: 100 μm. **(D–F)** The relative increasement of early apoptosis in irradiated cells between hypoxia and normoxia at 6 h **(D)**, 12 h **(E)** and 24 h **(F)** after radiation.

For the evaluation of mouse embryonic fibroblast cell performance under ultra-high dose rate FLASH irradiation, we assessed the apoptosis proportion of cyt c-normal cells at 6, 12, and 24 h after the irradiation by Dead Cell Apoptosis Kit with Alexa Fluor 488 annexin V and PI. Cells with Hoechst+/annexin V+/ PI- or Hoechst+/annexin V+/PI+ were recognized as early apoptotic cells, late apoptotic and necrotic cells respectively, representive images were shown in (Figures 3A–C). As shown in Supplementary Figure 1, compared with the unirradiated control, the relative early apoptosis level of irradiated normal fibroblast cells was increased with time in both hypoxic and normoxic conditions, and the proportion of early apoptosis at 6 h was relatively low (Supplementary Figures 1A,B). For the comparison of the irradiated cells in hypoxia and normoxia, the early apoptosis level in hypoxia was significantly lower than that of normoxia, with 0.73, 0.76, and 0.63-fold of the normoxic group at 6, 12, and 24 h, respectively (Figures 3D–F). And the overall proportion of early apoptosis of irradiated cells in the hypoxic condition was obviously lower than that of the normoxic condition (Supplementary Figures 1A,B).

**FIGURE 4:**
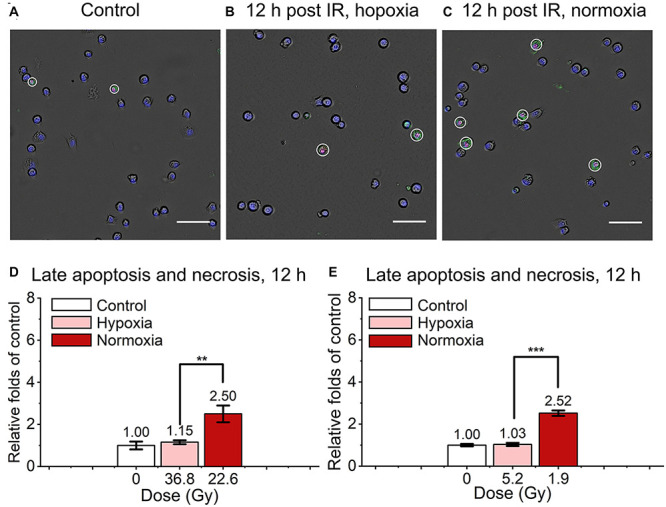
Late apoptosis and necrosis of irradiated normal fibroblast cells after FLASH irradiation. **(A–C)** Representative images of control **(A)**, irradiated cells in hypoxic **(B)** and normoxic condition **(C)** at 12 h after radiation. Late apoptotic and necrotic cells were marked with white circles. Scale bars: 100 μm. **(D,E)** The relative increasement of late apoptosis and necrosis in irradiated cells between hypoxia and normoxia at 12 h after high **(D)** or low **(E)** doses of radiation.

We also detected and analyzed the late apoptosis and necrosis level of irradiated normal fibroblast cells after FLASH-IR, as shown in Figure 4. Similar with the results of early apoptosis, the relative late apoptosis and necrosis level of irradiated cells were increased with time in both hypoxic and normoxic conditions (Supplementary Figures 1C,D). And compared with the irradiated group in normoxic condition, the irradiated cells in hypoxic condition showed significant lower level of late apoptosis and necrosis at 12 h, both after high doses and low doses irradiaion (Figures 4D,E). However, at 24 h, irradiated cells reached high level of late apoptosis and necrosis but without significant difference between them in both hypoxic and normoxic conditions (Supplementary Figures 2A,B).

### Cyt c-Null Mouse Embryonic Fibroblast Cells Show Lower Apoptosis Proportion After FLASH Irradiation

**FIGURE 5:**
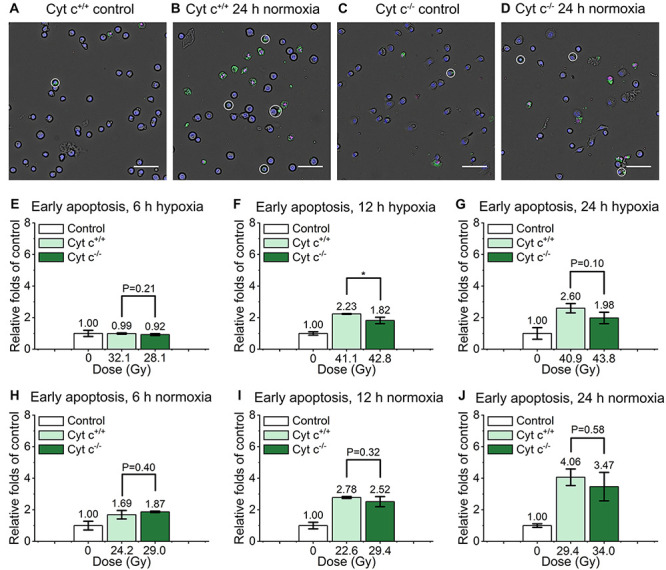
Comparison of early apoptosis between irradiated cyt c^+/+^ and cyt c^–/–^ cells after FLASH irradiation. **(A–D)** Representative images of cyt c^+/+^ control **(A)**, cyt c^+/+^ irradiated **(B)**, cyt c^–/–^ control **(C)** and cyt c^–/–^ irradiated cells **(D)** at 24 h in normoxic condition. Early apoptotic cells were marked with white circles. Scale bars: 100 μm. **(E–J)** The relative increasement of early apoptosis for irradiated cyt c^+/+^ and cyt c^–/–^ cells at 6 h in hypoxia **(E)**, 6 h in normoxia **(H)**, 12 h in hypoxia **(F)**, 12 h in normoxia **(I)**, 24 h in hypoxia **(G)** and 24 h in normoxia **(J)**.

As shown in the Figure 5, the relative early apoptosis level of irradiated normal (cyt c^+/+^) and cyt c-null (cyt c^–/–^) cells at different time and condition was compared. There was slightly decrease of early apoptosis proportion of cyt c^–/–^ cells at 6 h hypoxia (Figure 5E and Supplementary Figure 3A), 12 h in hypoxia (Figure 5F and Supplementary Figure 3B), and 24 h in normoxia (Figure 5J and Supplementary Figure 3F) compared with relative cyt c^+/+^ cells in certain conditions. The rest of the groups show similar relative early apoptosis level between irradiated cyt c^+/+^ and cyt c^–/–^ cells (Figures 5G–I and Supplementary Figures 3AC–E), showing no obvious difference of FLASH irradiation induced early apoptosis between cyt c^+/+^ and cyt c^–/–^ cells.

**FIGURE 6:**
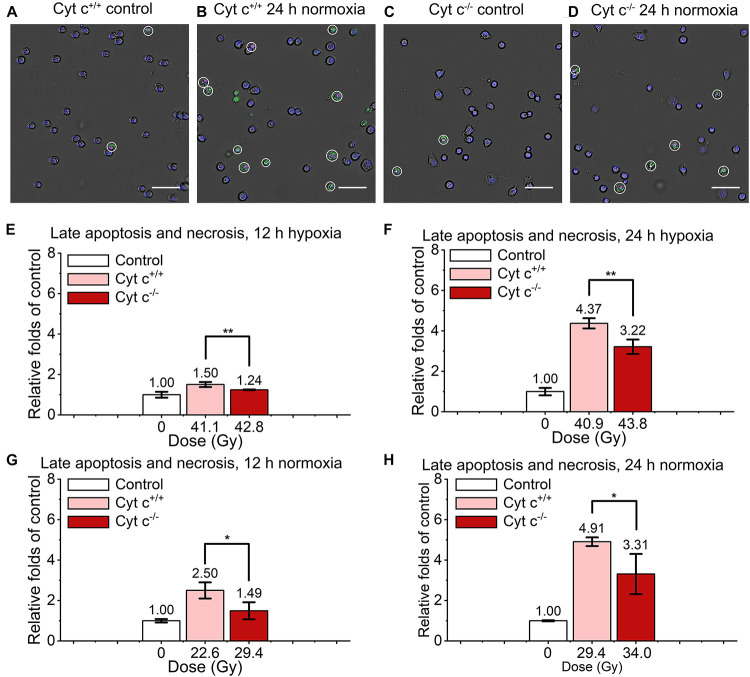
Comparison of late apoptosis and necrosis between irradiated cyt c^+/+^ and cyt c^–/–^ cells after FLASH irradiation. **(A–D)** Representative images of cyt c^+/+^ control **(A)**, cyt c^+/+^ irradiated **(B)**, cyt c^–/–^ control **(C)** and cyt c^–/–^ irradiated cells **(D)** at 24 h in normoxic condition. Late apoptotic and necrosis cells were marked with white circles. Scale bars: 100 μm. **(E–H)** The relative increasement of late apoptotic and necrosis for irradiated cyt c^+/+^ and cyt c^–/–^ cells at 12 h in hypoxia **(E)**, 12 h in normoxia **(G)**, 24 h in hypoxia **(F)**, and 24 h in normoxia **(H)**.

The comparison of relative late apoptosis and necrosis level of irradiated cyt c^+/+^ and cyt c^–/–^ cells at different time were shown in Figure 6. The difference of late apoptosis and necrosis proportion between irradiated cyt c^+/+^ and cyt c^–/–^ cells is significant at 12 h in hypoxia (Figure 6E), 12 h in normoxia (Figure 6G), 24 h in hypoxia (Figure 6F) and 24 h in normoxia (Figure 6H), and the relative late apoptosis and necrosis level of irradiated cyt c^–/–^ cells were 0.83, 0.60, 0.74 and 0.67 folds of irradiated cyt c^+/+^ cells in certain conditions, respectively, indicating mitochondrial dysfunction enhanced radio-resistance of normal fibroblast cells with less late apoptosis and necrosis after FLASH irradiation. And similar results of cyt c^–/–^ cells were also shown when receiving lower doses of FLASH irradiation (Supplementary Figures 4A,B).

### Apoptosis and Caspase-3/7 Detection After Conventional Irradiation

As shown in Supplementary Figure 6, the irradiated normal fibroblast cells in hypoxia showed lower early apoptosis level as well as late apoptosis and necrosis level compared with normoxia after conventional irradiation. The difference between hypoxia and normoxia is significant at 6 h (Supplementary Figure 6A), 12 h (Supplementary Figures 6B,D) and 24 h (Supplementary Figures 6C,E), indicating the radio-resistance enhancement of cyt c^+/+^ cells at hypoxic condition.

The caspase-3/7 chemiluminescence also revealed significant lower level at hypoxia compared with normoxia at 24 h after irradiation for cyt c^+/+^ cells (Supplementary Figure 8A) and cyt c^–/–^ cells (Supplementary Figure 8B). Besides, compared with conventional irradiation, FLASH irradiation showed high overall level of relative increment of early apoptosis, late apoptosis and necrosis, especially at 24 h both hypoxic and normoxic condition.

The comparison of relative apoptosis level of irradiated cyt c^+/+^ and cyt c^–/–^ cells at different time or condition were shown in Supplementary Figure 7. There was certain decrease of early apoptosis proportion of cyt c^–/–^ cells at all time points, in which the difference was significant at 6 h normoxia (Supplementary Figure 7D), 12 h hypoxia (Supplementary Figure 7B), 24 h hypoxia (Supplementary Figure 7C) and 24 h normoxia (Supplementary Figure 7F) compared with relative cyt c^+/+^ cells.

Similarly, the difference of late apoptosis and necrosis proportion between irradiated cyt c^+/+^ and cyt c^–/–^ cells is significant at 12 h in hypoxia (Supplementary Figure 7G), 12 h in normoxia (Supplementary Figure 7H), 24 h in hypoxia (Supplementary Figure 7I) and 24 h in normoxia (Supplementary Figure 7J), indicating the crucial role of mitochondrial function in apoptosis of normal fibroblast cells induced by radiation.

As shown in Supplementary Figures 8C,D, caspase-3/7 level of both cyt c^+/+^ and cyt c^–/–^ cells were low at hypoxia, caspase-3/7 level of cyt c^–/–^ cells decreased slightly compared with cyt c^+/+^ cells but the difference was not significant. At normoxia, the caspase-3/7 level of cyt c^–/–^ cells was significantly lower compared with cyt c^+/+^ cells.

## Discussion

In this article, we revealed early apoptosis, late apoptosis and necrosis induced by FLASH irradiation in both cyt c^+/+^ and cyt c^–/–^ mouse embryonic fibroblast cells. The apoptosis proportion of cyt c^+/+^ cell was significantly lower in hypoxia than that of in normoxia, indicating the increased resistance of normal fibroblast cell after FLASH irradiation in hypoxia. The late apoptosis and necrosis proportion of irradiated cyt c^–/–^ was significantly lower in both hypoxia and normoxia compared with irradiated normal cyt c^+/+^ cells, indicating mitochondrial dysfunction enhanced radio-resistance of mouse embryonic fibroblast cells after FLASH irradiation.

Due to the shot-to-shot fluctuations during laser acceleration, doses of laser-driven ion beams were different in every shot of irradiation and it is hard to keep it constant. Besides, the assay used to detect apoptosis and necrosis was an endpoint method so the doses were not same in experiments. We tried to use similar doses in the comparison group or choose the low doses for high apoptosis level. Although the doses were not completely consistent, the lower doses showed a higher value, indicating that the conclusion was reliable.

The normal tissue protection and tumor growth delay in animal models under FLASH irradiation suggested a potential role for FLASH in treating humans (Vozenin et al., 2019b). Our results showed that the apoptosis level of normal fibroblast cell was significantly lower in hypoxia compared with that in normoxia. Given the known role of oxygen in modulating radio-sensitivity, it is rationalized that FLASH irradiation could cause a rapid consumption of local oxygen that is much faster than tissue reoxygenation kinetics (Vozenin et al., 2019b). The types of cell death after radiation depend on a number of factors including cell type, radiation dose and quality, oxygen tension and so on (Sia et al., 2020). Oxygen tension modulates cell death after irradiation, with reduction in chromosomal aberrations of cells irradiated in hypoxic conditions (Sia et al., 2020). And it is presumed to related to kinetic competition between the oxygen ‘fixation’ of DNA damage and chemical repair processes (Sia et al., 2020).

Mitochondria plays crucial part in cell fate after irradiation. The mitochondria apoptotic pathway is the principal pathway of cell death after conventional low dose rate irradiation (Sinha et al., 2013; Li et al., 2019), in which cyt c serves as key component of apoptosis initiator (Jiang and Wang, 2004). The release of cyt c from mitochondria in response to multiple apoptotic stimuli can induce a series of biochemical responses leading to caspase activation and subsequent cell death (Jiang and Wang, 2004). There have been reports that mitochondrial permeabilization is generally more closely linked to events of late apoptosis and necrosis (Kinnally et al., 2011). And cells lacking cyt c show reduced caspase-3 activation and are resistant to the proapoptotic effects of UV irradiation, serum withdrawal, or staurosporine (Li et al., 2000). In our results, late apoptosis and necrosis proportion of irradiated cyt c^–/–^ cells showed significant decrease in both hypoxia and normoxia compared with irradiated normal cyt c^+/+^ cells, indicating that the deficiency of cyt c in mouse embryonic fibroblast cells also reduced the proportion of mitochondrial-mediated apoptosis under FLASH-IR. In addition, functional changes in mitochondria in response to radiation exposure could influence epigenetic parameters and genome stability (Baulch, 2019). And cyt c independent ceramide apoptotic pathway is also related to mitochondria. Research showed that by regulating mitochondrial function, DDR pathway and MAPK pathway, ceramide could potentially mediated gamma radiation-induced apoptosis (Yang et al., 2021). Presently we cannot exclude the impact of the apoptosis in cyt c independent apoptotic pathway after FLASH irradiation. More validating experiments need to be further performed.

The ultra-high dose rates and ultra-short irradiation time implemented by laser accelerators is promising, but the instability in different shots is also a concern (Lundh et al., 2012; Manti et al., 2017), which is sure to impede the interpretation and repetition of FLASH experimental results. The more stable, easily controlled and widely applicable FLASH irradiation as well as more biological experiments for scientific and clinical use are further expected.

## Data Availability Statement

The original contributions presented in the study are included in the article/Supplementary Material, further inquiries can be directed to the corresponding author/s.

## Author Contributions

GY, WM, and XY conceived the research plan. JH, CLu, and JQ designed and performed the biology experiments, analyzed results, and produced figures. ZM, WM, and GY designed and constructed the irradiation setup. ZM, YL, ZP, DK, SX, ZL, YGa, GQ, YS, SC, ZC, YZ, and YGe supervised by WM performed the laser acceleration and cell irradiation experiments. ZM and YL measured the proton radiation and provided the dose data. JH wrote the initial draft of the manuscript. GY wrote the final draft of the manuscript. All authors commented on the manuscript.

## Conflict of Interest

The authors declare that the research was conducted in the absence of any commercial or financial relationships that could be construed as a potential conflict of interest.

## Abbreviations

Cyt c: cytochrome c
FBS: fetal bovine serum
PI: propidium iodide
RT: radiotherapy
FLASH-IR: FLASH irradiation
CLAPA: compact laser plasma accelerator system
FWHM: full width at half maximum
TPS: Thomson parabola spectrometer
RCF: radiochromic film.
