# Supplementary material for: Ultra-High Dose Rate FLASH Irradiation Induced Radio-Resistance of Normal Fibroblast Cells Can Be Enhanced by Hypoxia and Mitochondrial Dysfunction Resulting From Loss of Cytochrome C
Source: Front Cell Dev Biol. 2021 Apr 30;9:672929. doi: 10.3389/fcell.2021.672929 (PMC8121317; doi:10.3389/fcell.2021.672929)
Supplement: Supplementary file 1 [file Data_Sheet_1.pdf]

Supplementary Material

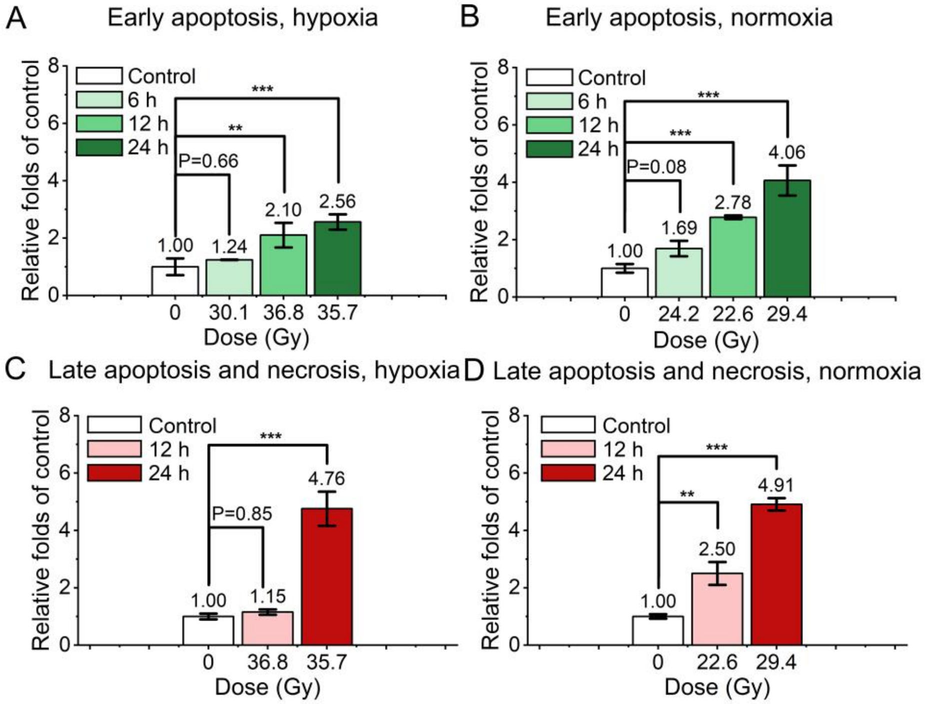

Supplementary Figure 1. Early apoptosis, late apoptosis and necrosis of normal fibroblast cells after FLASH irradiation. **(A-B)** The relative increasement of early apoptosis for cells at 6, 12 and 24 h after radiation in hypoxic (A) and normoxic condition (B). **(C-D)** The relative increasement of late apoptosis and necrosis for cells at 12 and 24 hours after radiation in hypoxic (C) and normoxic condition(D).

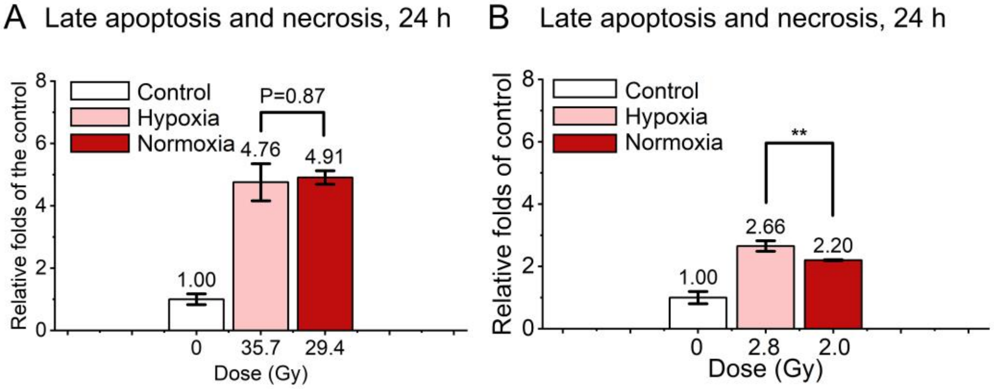

Supplementary Figure 2. Late apoptosis and necrosis of normal fibroblast cells after FLASH irradiation. **(A-B)** The relative increasement of late apoptosis and necrosis in irradiated cells between hypoxia and normoxia at 24 h after high (A) or low (B) doses of irradiation.

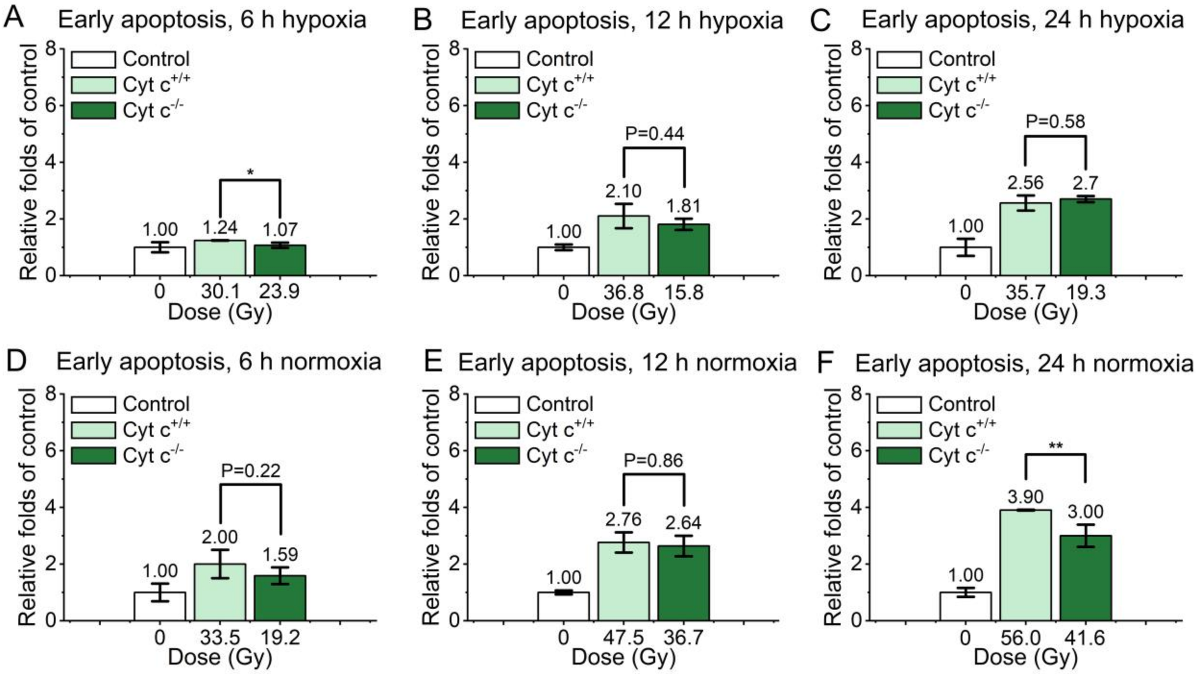

Supplementary Figure 3. Comparison of early apoptosis between irradiated cyt $c^{+/+}$ and cyt $c^{-/-}$ cells after FLASH irradiation. (**A-F**) The relative increasement of early apoptosis for irradiated cyt $c^{+/+}$ and cyt $c^{-/-}$ cells at 6 h in hypoxia (A), 6 h in normoxia (D), 12 h in hypoxia (B), 12 h in normoxia (E), 24 h in hypoxia (C) and 24 h in normoxia (F).

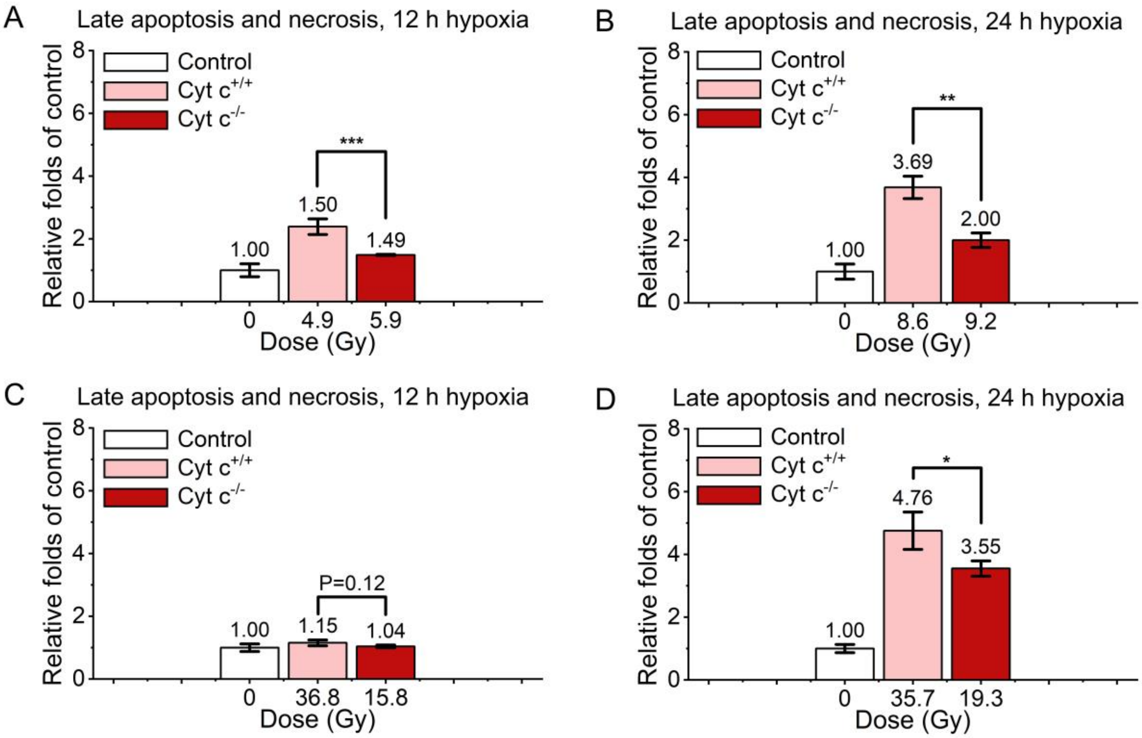

Supplementary Figure 4. Comparison of late apoptosis and necrosis between irradiated cyt $c^{+/+}$ and cyt $c^{-/-}$ cells after FLASH irradiation. **(A-B)** The relative increasement of late apoptosis and necrosis for irradiated cyt $c^{+/+}$ and cyt $c^{-/-}$ cells at 12 h in hypoxia (A), 24 h in hypoxia (B). **(C-D)** The relative increasement of late apoptosis and necrosis for irradiated cyt $c^{+/+}$ and cyt $c^{-/-}$ cells at 12 h in hypoxia (C), 24 h in hypoxia (D).

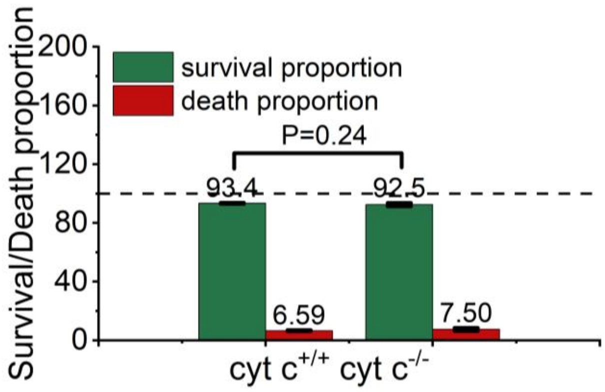

Supplementary Figure 5. Viability of cyt $c^{+/+}$ and cyt $c^{-/-}$ cells. Cells were stained with Calcin-AM and PI.

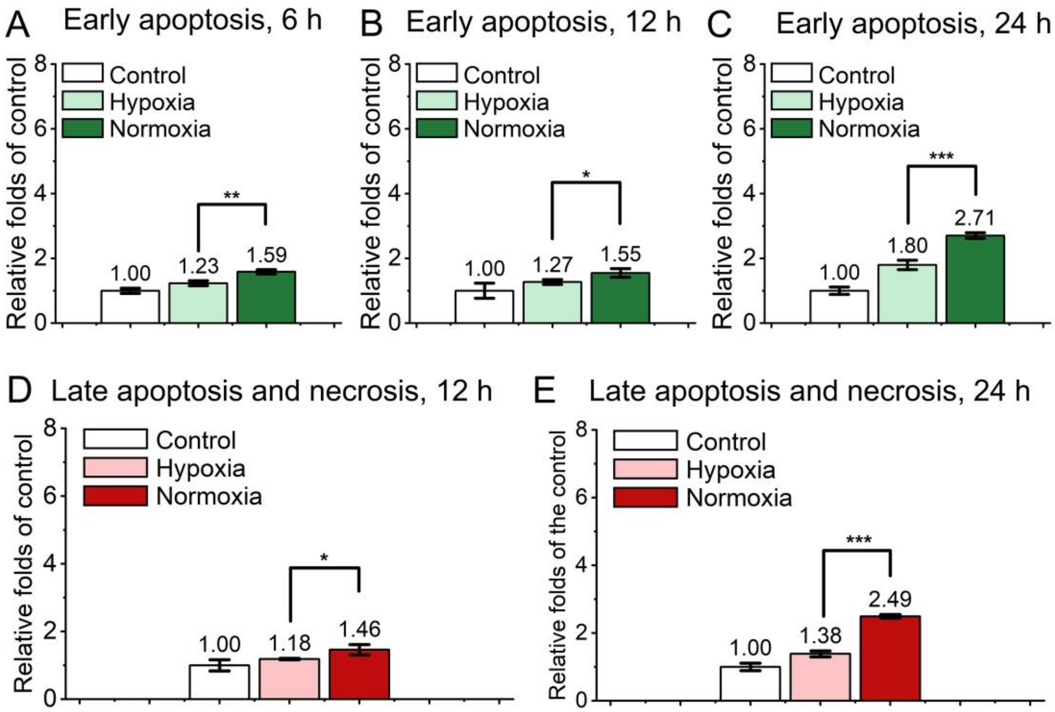

Supplementary Figure 6. Early apoptosis, late apoptosis and necrosis of irradiated normal fibroblast cells after conventional irradiation. **(A-C)** The relative increasement of early apoptosis in irradiated cells between hypoxia and normoxia at 6 h (A), 12 h (B) and 24 h (C) after radiation. **(D-E)** The relative increasement of late apoptosis and necrosis in irradiated cells between hypoxia and normoxia at 12 h (D) and 24 h (E) after radiation.

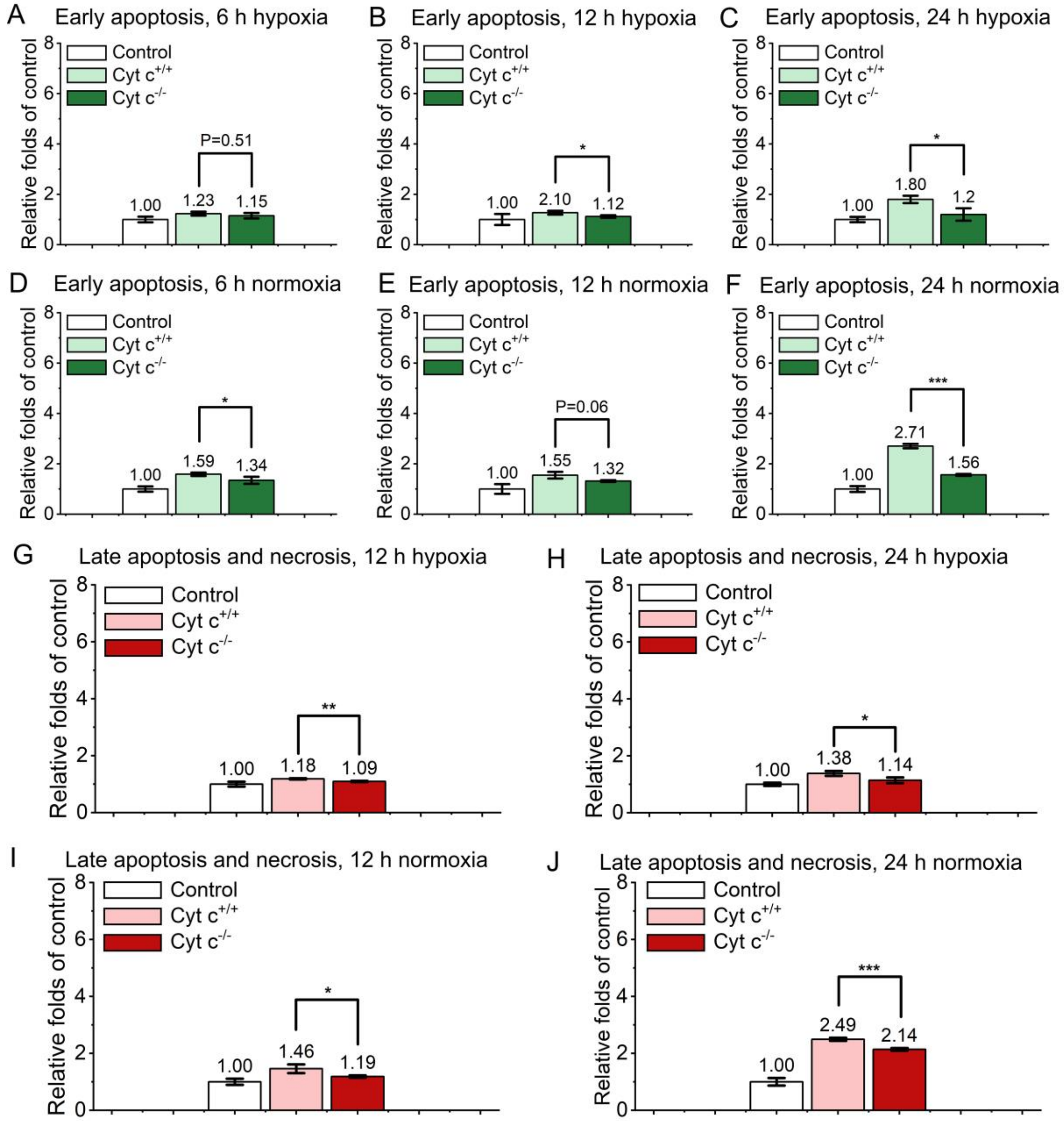

Supplementary Figure 7. Comparison of early apoptosis, late apoptosis and necrosis between irradiated cyt $c^{+/+}$ and cyt $c^{-/-}$ cells after conventional irradiation at the deose of 30 Gy. **(A-F)** The relative increasement of early apoptosis for irradiated cyt $c^{+/+}$ and cyt $c^{-/-}$ cells at 6 h in hypoxia (A), 6 h in normoxia (D), 12 h in hypoxia (B), 12 h in normoxia (E), 24 h in hypoxia (C) and 24 h in normoxia (F). **(G-J)** The relative increasement of late apoptosis and necrosis for irradiated cyt $c^{+/+}$ and cyt $c^{-/-}$ cells at 12 h in hypoxia (G), 12 h in normoxia (I), 24 h in hypoxia (H), and 24 h in normoxia (J).

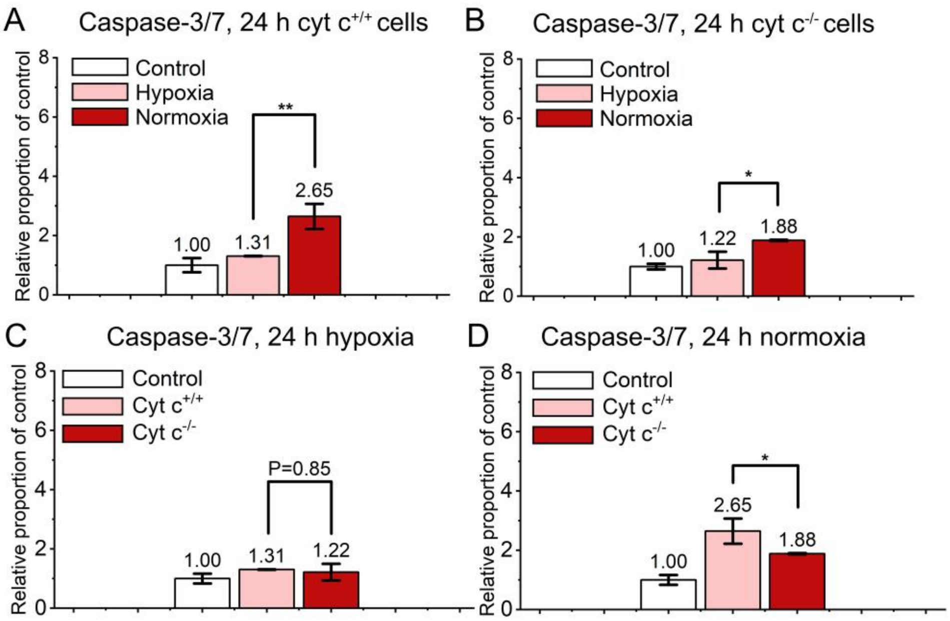

Supplementary Figure 8. Caspase-3/7 level of irradiated cyt $c^{+/+}$ and cyt $c^{-/-}$ cells under hypoxia and normoxia at 24 h after conventional irradiation at the dose of 30 Gy. **(A-B)** The relative proportion of caspase-3/7 level between hypoxia and normoxia of cyt $c^{+/+}$ (A) and cyt $c^{-/-}$ (B) cells. **(C-D)** The relative increasement of caspase-3/7 level for irradiated cyt $c^{+/+}$ and cyt $c^{-/-}$ cells at 24 h in hypoxia (C) and 24 h in normoxia (D).
